# KDEL Receptors: Pathophysiological Functions, Therapeutic Options, and Biotechnological Opportunities

**DOI:** 10.3390/biomedicines10061234

**Published:** 2022-05-25

**Authors:** Ilaria Cela, Beatrice Dufrusine, Claudia Rossi, Alberto Luini, Vincenzo De Laurenzi, Luca Federici, Michele Sallese

**Affiliations:** 1 Department of Innovative Technologies in Medicine and Dentistry, “G. D’Annunzio” University of Chieti-Pescara, 66100 Chieti, Italy; ilaria.cela@unich.it (I.C.); beatrice.dufrusine@unich.it (B.D.); vincenzo.delaurenzi@unich.it (V.D.L.); luca.federici@unich.it (L.F.); 2 Center for Advanced Studies and Technology (CAST), “G. D’Annunzio” University of Chieti-Pescara, 66100 Chieti, Italy; claudia.rossi@unich.it; 3 Department of Psychological, Health and Territorial Sciences, “G. D’Annunzio” University of Chieti-Pescara, 66100 Chieti, Italy; 4 Institute for Experimental Endocrinology and Oncology “Gaetano Salvatore” (IEOS)-Branch Office, Via Pietro Castellino 111, 80131 Napoli, Italy; a.luini@ieos.cnr.it

**Keywords:** endoplasmic reticulum homeostasis, chaperones retrieval, unfolded protein response, viruses trafficking, targeted therapies

## Abstract

KDEL receptors (KDELRs) are ubiquitous seven-transmembrane domain proteins encoded by three mammalian genes. They bind to and retro-transport endoplasmic reticulum (ER)-resident proteins with a C-terminal Lys-Asp-Glu-Leu (KDEL) sequence or variants thereof. In doing this, KDELR participates in the ER quality control of newly synthesized proteins and the unfolded protein response. The binding of KDEL proteins to KDELR initiates signaling cascades involving three alpha subunits of heterotrimeric G proteins, Src family kinases, protein kinases A (PKAs), and mitogen-activated protein kinases (MAPKs). These signaling pathways coordinate membrane trafficking flows between secretory compartments and control the degradation of the extracellular matrix (ECM), an important step in cancer progression. Considering the basic cellular functions performed by KDELRs, their association with various diseases is not surprising. KDELR mutants unable to bind the collagen-specific chaperon heat-shock protein 47 (HSP47) cause the osteogenesis imperfecta. Moreover, the overexpression of KDELRs appears to be linked to neurodegenerative diseases that share pathological ER-stress and activation of the unfolded protein response (UPR). Even immune function requires a functional KDELR1, as its mutants reduce the number of T lymphocytes and impair antiviral immunity. Several studies have also brought to light the exploitation of the shuttle activity of KDELR during the intoxication and maturation/exit of viral particles. Based on the above, KDELRs can be considered potential targets for the development of novel therapeutic strategies for a variety of diseases involving proteostasis disruption, cancer progression, and infectious disease. However, no drugs targeting KDELR functions are available to date; rather, KDELR has been leveraged to deliver drugs efficiently into cells or improve antigen presentation.

## 1. Introduction

KDEL receptors (KDELRs) are seven-transmembrane-domain proteins primarily involved in the retrieval of endoplasmic reticulum (ER)-resident proteins from post-ER compartments to the ER lumen; this is functionally important to the maintenance of proteostasis in the ER [1,2,3]. KDELRs were first described in yeast by Pelham’s lab, which identified two genes, ERD1 and ERD2, required for the retention within cells of a number of ER-chaperones, all bearing the characteristic C-terminal tetrapeptide motif HDEL [4,5]. However, while ERD1-deleted yeasts exhibited defects in the N-glycosylation of their secreted proteins [4], ERD2 appeared to be closely related to the retrieval of ER-proteins, eventually recognizing it as the yeast HDEL receptor (or Erd2p) [5,6].

After these findings, human ERD2 or KDELR1 was identified [7], rapidly followed by the identification of KDELR2, also known as ERD-like protein 1 (ELP1) [8,9]. A third receptor, known as KDELR3, was later characterized [10]. Human orthologs of the yeast HDEL receptor have been termed KDELRs since the KDEL-retrieval motif is the most common in mammals [1,7,11]. The three KDELRs have highly conserved sequences in terms of both identity and similarity, as well as approximately 70% similarity to yeast ERD2 [1].

**Figure 1:**
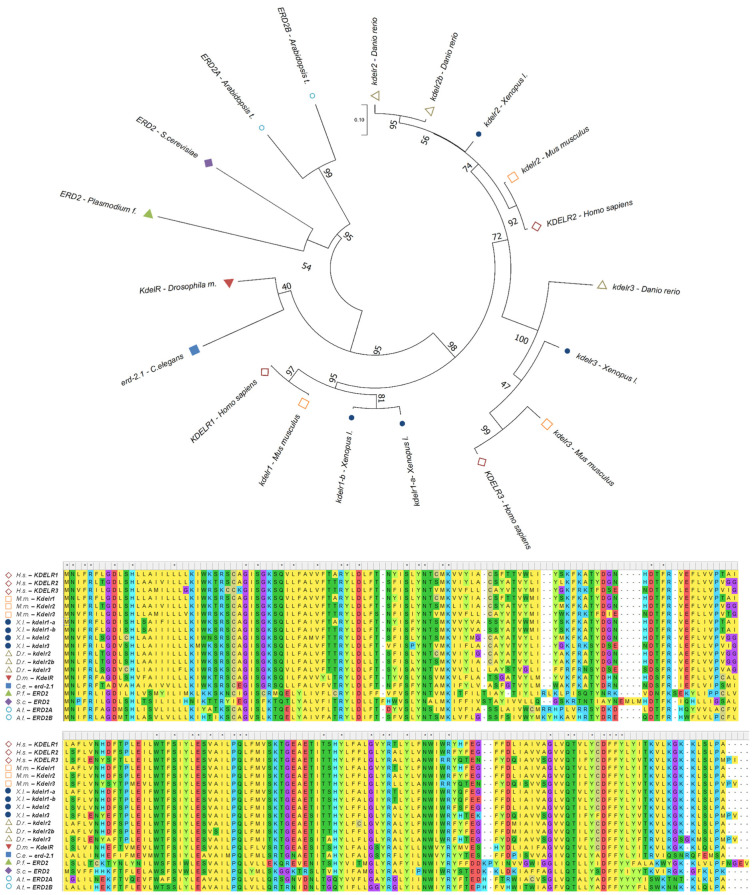
**H/KDEL receptors’ orthologues show high levels of conservation among different eukaryotic organisms.** Phylogenetic tree showing the evolutionary relationship of KDELRs in several eukaryotic organisms as inferred, from protein sequence alignments, using the maximum likelihood method in MEGA 11 software (version 11, USA) [18] and corrected using the Poisson model. The tree is drawn to scale, with branch lengths in the same units as those of the evolutionary distances used to infer the phylogenetic tree. The evolutionary distances are in the units of the number of amino acid substitutions per site. The branch support values were gained by bootstrapping method (500 iterations). Protein sequences of KDELRs retrieved from Uniprot (Uniprot release 2022_01, Uniprot Consortium) [19] were aligned by ClustalW on MEGA 11 software (version 11, USA) [18]. Asteriks indicate conserved amino acids within sequences. H.s., *Homo sapiens*; M.m., *Mus musculus*; X.l., *Xenopus laevis*; D.r., *Danio rerio*; D.m., *Drosophila melanogaster*; C.e., *Caenorhabditis elegans*; P.f., *Plasmodium falciparum*; S.c., *Saccharomyces cerevisiae*; A.t., *Arabidopsis thaliana*.

Over the years, H/KDEL receptor orthologues have been detected in other eukaryotic organisms, including a single KDELR gene in *Drosophila melanogaster* [12], *Caenorhabditis elegans* [13], and *Plasmodium falciparum* [14], several ERD2-like proteins in *Arabidopsis thaliana* [15,16], and three KDELRs genes in *Xenopus laevis* and *Danio rerio* (Figure 1) [12,17]. From an evolutionary perspective, the high levels of conservation suggest that strong selective pressure was applied to these genes to conserve their functions (Figure 1). More interestingly, in vertebrates, including humans, the KDELR gene underwent triplication, giving rise to functional redundancy with a certain degree of specificity among the three different isoforms (see below). Indeed, human KDELRs have been shown to have differential affinities for specific variants of the tetrapeptide ligand [17].

## 2. Physiological and Pathological Cell Functions of KDEL Receptors

In recent years, numerous studies have sought to elucidate the roles of KDELRs in mammalian cell physiology, and their possible involvement in human diseases [1,2,3,20].

Most of the chaperones and enzymes resident in the lumen of ER are retained despite membrane trafficking because they are organized in large multiprotein complex and actively sorted out from cargo proteins that are transported anterogradely [21]. However, a small fraction of these proteins may be found in post-ER compartments for several reasons that include their involvement in ER quality control, leakage of the sorting machinery, perturbations of calcium ion (Ca^2+^) homeostasis, or unfolded protein response (UPR)-induced overexpression. These chaperones, by virtue of the KDEL motif present in their C-terminal region, bind the KDELRs in the Golgi complex. KDELR-bound chaperones undergo conformational changes in order to expose their di-lysine motif to bind COPI coatomer. The chaperone-KDELR complex retro-translocates to the ER, within COPI-coated vesicles, where chaperones are released [22].

Recently, KDELRs were also found to regulate the maturation of the epithelial sodium channel (ENaC) by working in concert with the endoplasmic reticulum protein 29 kDa (ERp29). Removal of the C-terminal KEEL sequence from ERp29 or the KDELR1 itself impairs the functional expression of ENaC without changing the number of channels on the plasma membrane [23].

More recent studies have reported complex cycling of KDELRs between the plasma membrane and the Golgi, thereby passing through the RAB11- and RAB14-positive endosomal compartment [24].

**Figure 2:**
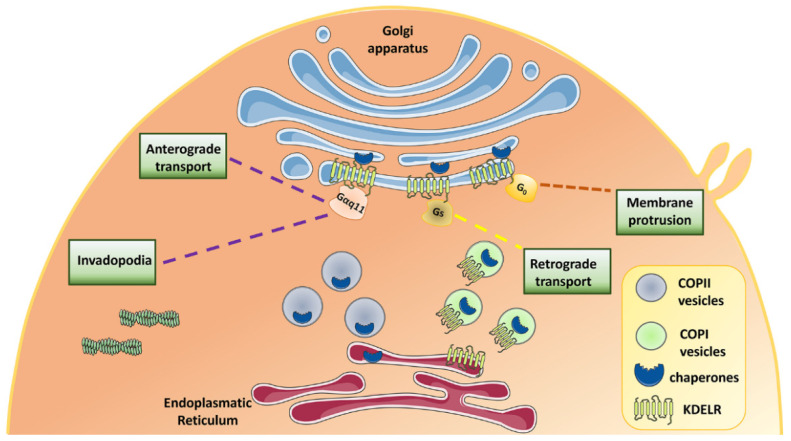
**Schematic illustration of the role of KDELRs in the control of multiple cellular functions.** KDELRs regulate the homeostasis of membrane transport between ER, Golgi, and plasma membrane via the Gq and Gs signaling pathways. KDELRs stimulate invadopodia formation via activation of Gq signaling.

Our pioneering work has shown that when Golgi-localised KDELRs are bound by chaperones, they initiate a signaling cascade involving the activation of Golgi-based Gαq/11 and Src family tyrosine kinases (SFKs), which in turn phosphorylate a number of proteins to modulate intra-Golgi trafficking [25,26] (Figure 2). More generally, one of the functions of this pathway is to provide a feed-forward circuit that controls cargo gating at the Golgi complex, preserving the dynamic equilibrium of this organelle during membrane trafficking [25,27] (Figure 2). Besides membrane trafficking, KDELR activation regulates invadopodia and focal adhesion dynamics. Specifically, KDELR1 and KDELR2, by activating SFKs, stimulate invadopodia formation and extracellular matrix (ECM) degradation, while KDELR3 is only marginally involved in this process [28] (Figure 2). In addition, KDELR promotes focal adhesion kinase (FAK) recruitment to invadopodia and phosphorylation on Y397 and Y861. From a functional standpoint, the phosphorylation of FAK Y397 is required for KDELR-stimulated ECM degradation [29].

Activated KDELR also couples to Gαs and activates adenylyl cyclase type 9 (AC9) and protein kinase A (PKA) [30]. This signaling cascade participates in the retrograde Golgi to ER trafficking and is important to balance transport fluxes between these organelles (Figure 2). Prohibitin (PHB) and acyl-CoA binding domain-containing 3 (ACBD3) scaffold proteins belong to the KDELR-PKA signalosome and take part in the retro-translocation of the KDELR [31,32]. To accomplish this task, KDELR-activated PKA phosphorylates numerous proteins involved in membrane trafficking, although many other PKA targets play a role in cell growth, motility, energy metabolism, and transcription [30].

Furthermore, KDELR can physically interact and activate Gαo on the Golgi membranes. Specifically, the Gαo pool localized on the plasma membrane regulates the formation of membrane protrusions. Instead, the Golgi pool of Gαo, which is activated by KDELRs, signals through the small GTPases Rab1/Rab3 and stimulates anterograde trafficking with the ultimate goal of delivering the material required for the growth and stabilization of membrane protrusions, such as neurites [33] (Figure 2).

KDELRs contribute to the maintenance of ER homeostasis and quality control of the ER primarily through the recovery of ER-resident proteins [34,35], but also by participating in the UPR [2], a cell reaction triggered by a wide array of cell stresses leading to the impairment of protein folding. Cells use three stress sensors, namely protein kinase RNA-like ER kinase (PERK), inositol-requiring enzyme 1 (IRE1), and activating transcription factor 6 (ATF6), located on ER membranes, to monitor homeostasis and activate a complex signaling and transcription program aimed at coping with stresses. Cells through the IRE1/X-box-binding protein 1 (XBP1) branch of the UPR upregulate KDELR2 and KDELR3 to deal with the increased burden of misfolded proteins and counteract the loss of ER-chaperones increased by the UPR [36,37]. KDELRs seem to also be involved in autophagy, an important mechanism strictly related to UPR and the clearance of misfolded cargoes [38,39]. In fact, the activation of KDELR1, but not KDELR2 and KDELR3, modulates the turnover of lipid droplets via autophagy and relocates lysosomes to a perinuclear region in order to sustain the secretory process [39]. KDELRs may control autophagy either through PKA signaling or through the classic mitogen-activated protein kinase (MAPK) cascade involving dual-specificity mitogen-activated protein kinase 1 (MEK1) and extracellular signal-regulated kinases (ERKs) [39,40].

## 3. Structural Features of KDEL Receptors

At first glance, the seven transmembrane (TM) domains of KDELRs together with their ability to bind and activate heterotrimeric G proteins might suggest that these receptors belong to the superfamily of G-protein-coupled receptors (GPCRs) [26,30,33]. However, in-depth sequence analysis indicated that KDELRs belong to the Pro-Gln-(PQ)-loop family of proteins [41,42]. Proteins belonging to this family, also known as SWEET, are often sugar transporters and are found in plants, mammals, and bacteria; however, some members were also shown to function as amino acid transporters (e.g., cystine and cationic amino acids) in lysosomes and vacuoles [22,41,42,43].

**Figure 3:**
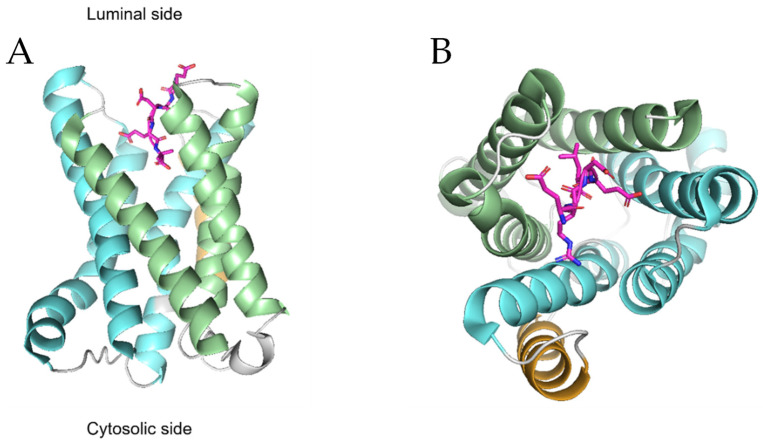
**Structural organization of KDELR.** The images show the side (**A**) and top view (**B**) of Gallus gallus KDELR2 according to the PDB database coordinates (PDB DOI: 10.2210/pdb6ZXR/pdb 27 April 2022). The two helical bundles made up of the first three and last three helices are shown in light green and light blue, respectively. The fourth transmembrane helix, which connects the two bundles, is shown in light brown. The RDEL ligand, represented in sticks, is located in the pocket formed by the TM1-3 and TM5-7 helices.

From a structural standpoint, recent studies on KDELRs [22,41,42,44,45] revealed that the first three transmembrane helices form a three-helix bundle (THB) arranged in the order of TM1-TM3-TM2, and the same arrangement is found in the last three helices (Figure 3); this overall organization is common to eukaryotic SWEET transporters [22]. The two THB domains are connected by TM4, with TM1 being juxtaposed to TM6 and TM2 to TM5. Within this structural organization, the N-terminal portion faces the luminal compartment (Golgi or ER), while the C-terminal portion faces the cytoplasm (Figure 3A), and the PQ doublet is located towards the end of TM5. Note that, unlike SWEET transporters, KDELRs retained only one PQ motif during evolution [41]. The receptor appears to be embedded in the phospholipid bilayer in a slightly asymmetrical position, with the cytosolic surfaces protruding outside the membrane towards the intracellular compartment, while the luminal portion, towards the ER or Golgi, is flush with the membrane [22]. A large polar cavity is present on the luminal side of the receptor with an electrostatically charged surface constituted by the side chains of TM1-3 and TM5-7. Specifically, there are a few cationic amino acids facing those with negative charges (e.g., R5-TM1 and R169-TM6 are opposite to E117-TM5 and D177-TM7). This luminal pocket serves as a recognition and binding site for the KDEL tetrapeptide of ER chaperones, the natural ligands for these receptors. The prominent cytosolic face of the receptor serves instead as a binding site for COP-I and COP-II, which assist receptor cycling between the ER and the Golgi [22].

These structural studies, based on chicken KDELR2, have also shed more light on the pH-dependent binding mechanism of KDEL carrier proteins to KDELR [44,45,46]. KDELR binds to its ligand and forms a stable complex in the Golgi lumen where the pH is acidic (pH 6.2). This chaperone-bound receptor recruits COP-I and initiates retrograde trafficking to the ER where the near-neutral pH (pH 7.2–7.4) of the lumen promotes the release of the ligand. At this point, the empty receptor returns to the Golgi via COP-II vesicles [45]. These mechanisms rely on a conserved histidine residue (H12) on TM1, which, due to its protonable nature, is considered the pH sensor for the retrieval of KDEL proteins. When the receptor is in the Golgi and is bound with KDEL proteins, H12 is protonated, and this is essential for the formation of a short hydrogen bond (SHB) between Y158 (TM6) and E127 (TM5), to which H12 itself is adjacent. SHB blocks the C-terminal portion (position −1) of the KDEL-peptide within the receptor after it has been committed to the luminal portion of the KDELR in a stepwise process, known as a handover pattern, whereby the peptide is initially captured by R169, then transferred to R5, then to R47, and finally blocked by SHB [44]. As mentioned earlier, many variants of the tetrapeptide binding motif are present on human ER-resident proteins [17,44]. Structural and functional studies have shown that the −1 and −2 positions within the canonical binding sequence are the most important for interaction with the KDELR, likely because the −1 and −2 positions can interact with as many as 10 and 6 amino acids of the receptor, respectively [47]. On the other hand, the −3 and −4 positions of the motif contact only 3 and 4 amino acids of the KDELR, and, indeed, the substitution of these amino acids produces less dramatic effects on receptor binding [47]. ER-resident proteins carrying divergent amino acids at the −3 and −4 positions, especially those lacking positively charged residues at the −4 position, which normally contact amino acids D50 and E117, can still bind the KDELR because their loss is compensated for by the positively charged amino acids present further upstream, for example at the −5 or −6 position. Indeed, the −5, −6, and −7 positions on the C-terminus of KDELR binding proteins, although less important than the last four positions, are able to interact with the receptor and contribute to binding [47]. All three KDELR isoforms exploit the upstream positions of the KDEL motif to bind their natural ligands. For instance, by using prototypical protein ligands, it was shown that a threonine at position −7 can interact with D112 of the receptor, while isoleucine at position −6 can interact with four amino acids of the receptor including I56 and L116 [47].

Once bound to the KDEL peptide, the receptor undergoes a conformational change based on the detachment of TM7 from TM5, resulting in the cytoplasmic exposure of a C-terminal lysine-rich group (K201, K204, and K206) that functions as a COP-I binding site [22]. In the ER, the higher pH causes the deprotonation of H12 and thus the destabilization of SHB, consequently inducing the release of the KDEL-peptide from the receptor [44]. Upon resuming the apo state, TM7 again bundles with TM5, and a negatively charged acidic motif is exposed on the cytosolic surface, consisting of several residues such as D87, E143, E145, and on the C-terminal of TM7 that are highly conserved in mammalian KDELRs. Interestingly, mutagenesis of these residues led to the retention of KDELRs within the ER, suggesting that this portion might be a kind of ER-exit motif and/or the specific binding site for COP-II [22,48].

## 4. Involvement of KDELRs in Human Diseases

As can be expected, the disturbance of ER balance along with alterations in the KDELR-retrieval pathway may result in detrimental effects, ultimately leading to several pathological conditions (Table 1). KDELRs were found upregulated/relocated in the ER in cerebral and myocardial ischemia [49,50,51], diabetes [52,53], and neurodegenerative diseases including Parkinson’s disease, Huntington’s disease, Pelizaeus–Merzbacher disease, as well as in amyotrophic lateral sclerosis (ASL) [35,40,54,55,56]. These neurodegenerative diseases share pathological ER stress leading to the accumulation of protein aggregates [40,54] and activation of the UPR, although aberrant autophagy has also been involved [57]. Upregulation of KDELRs could potentially be useful in neurodegenerative disease and after an ischemic attack to retain ER proteins and ensure the protective effects exerted by factors such as cardiomyokine cerebral dopamine neurotrophic factor (CDNF) or mesencephalic astrocyte-derived neurotrophic factor (MANF) that bind KDELR itself on the plasma membrane [58,59].

The role of KDELR in diabetes has not yet been elucidated; its association with the disease is based on receptor overexpression [52,53], its contribution to UPR [2], and its interaction with PHB and MANF [31,59]. It is also worthwhile to note that the IRE1/XBP1 pathway appears to be involved in insulin metabolism in pancreatic cells [67] while PHB, a scaffold protein that assists in the activation of KDELR-dependent Src for the regulation of membrane trafficking [31], induces diabetes in transgenic mice [68,69]. Finally, MANF is a protective factor for diabetes, and PHB knockout mice develop the disease [68,70].

Regarding KDELRs and immune responses, a recent study demonstrated that a mutation in KDELR1 disrupts the interaction between the receptor and protein phosphatase 1 (PP1), a key player in the regulation of T-cell homeostasis in response to cell stress [60]. Mice expressing this KDELR1 mutant had an increased rate of both CD4+ and CD8+ T lymphocyte apoptosis and a subsequent reduction in the number of naïve T-cells [60]. Another research group studying the effects of a different KDELR1 mutation confirmed T-cell lymphopenia and showed compromised antiviral immunity [61]. Although further examination is needed, so far, only KDELR1 has been found to be implicated in immune processes, thus proposing a putative functional specialization for this receptor isoform. The importance of KDELR1 in the immune response is also supported by mutations in the membrane trafficking partners of KDELRs. Homozygous mutation in the γ1 subunit of coat protein complex I (COPI; γ1-COP) that disrupts the binding of COPI to KDELRs impairs the retro-translocation of KDEL-bearing chaperones and causes combined immunodeficiency (CID) characterized by defective innate and adaptive immunity [71].

Bi-allelic variants of the KDELR2 gene have recently been described in patients with osteogenesis imperfecta (OI), a rare heterogeneous connective tissue disorder characterized by susceptibility to bone fractures along with neurodevelopmental disorders [62,63]. KDELR2-related osteogenesis imperfecta results from the inability of the collagen-chaperone, heat shock protein 47 (HSP47), to bind KDELR2; this prevents the dissociation of HSP47 from type 1 collagen. Therefore, HSP47 is not retro-translocated into the ER but is secreted together with collagen, impairing the formation of proper extracellular collagen fibers [62,63].

Finally, several studies described the involvement of KDELRs in the development and progression of cancer [64,65,66,72,73,74,75,76], but this aspect will not be fully addressed here because it deserves extensive and dedicated discussion. In short, interfering with KDELR1, KDELR2, and KDELR3 was found to reduce leukemic cell proliferation and hinder glioblastoma growth and invasiveness of metastatic melanoma, respectively [64,65,66,72,73,74]. These findings repurpose a specialization among the three KDELR isoforms and suggest that as yet unknown signaling pathways could be exploited by KDELRs. Indeed, the molecular mechanisms that KDELRs put in place to control cell proliferation and motility are only partially known and range from cytoskeletal reorganization [28,29] to the activation of the mechanistic target of rapamycin complex 1 (mTORC1) pathway [65]. We believe that KDELRs are used to regulate so many functions because they are ideally placed to work as a hub that senses the metabolic state of the ER and signals to other cellular machinery that will adapt their activities accordingly.

## 5. Role of KDELR in Intoxication and Viral Infection

**Figure 4:**
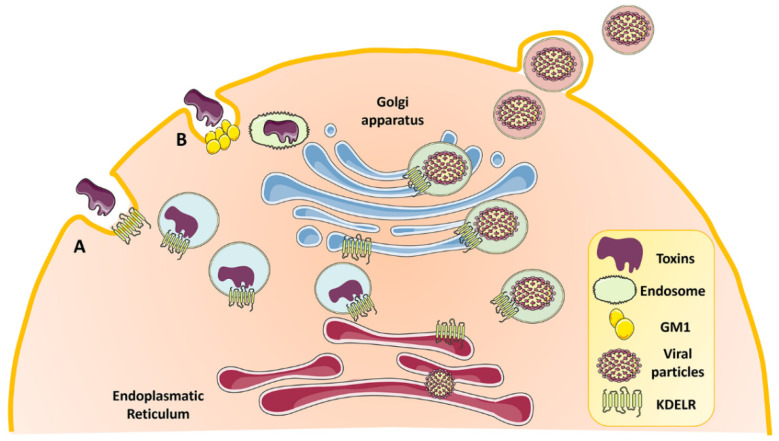
**KDELR is involved in the internalization, trafficking, and secretion of toxins and viral particles.** (**A**) Surface-expressed KDELRs can carry the internalization of toxins. (**B**) Cholera toxin (CTX) can be internalized by endocytosis after binding to GM1 ganglioside. After uptake, the A subunit of CTX dissociates and is transported to the Golgi and ER. Retrograde transport of the toxin from the Golgi to the ER is mediated by KDELRs through COPI vesicles. Furthermore, KDELRs are involved in the intracellular trafficking and maturation of viral particles.

Several works have brought to light the exploitation of KDELR in the membrane trafficking of toxins and different types of viruses [77,78,79,80,81,82,83,84,85] (Figure 4). The yeast killer toxin K28 [78], the cholera toxin A fragment [77], the thermolabile LT toxin of *E. coli* [77], and the Pseudomonas exotoxin A [79,80] were found to hijack the KDELR retrieval pathway for efficient transport from the Golgi to the ER. This shuttling mechanism is made possible by the presence of a KDEL or KDEL-like motif at the C-terminus of protein toxins [1,86]. Interestingly, KDELR-mediated transport of toxins might involve not only the early secretory pathway, but might extend to the plasma membrane, likely to contribute to toxin internalization, where KDELRs have been found both in yeast and mammalian cells [24,58,87,88,89]. For example, transport of the yeast killer toxin K28 exploits the KDELR present on the yeast plasma membrane [78].

KDELRs have also been characterized as intracellular receptors for virus trafficking within the host cells (Figure 4). They contribute to the maturation of viral particles and their consequent egress in the case of the Dengue virus [81], vaccinia viruses [82], and Japanese encephalitis virus [83]. In the case of Dengue viruses, the viral precursor membrane protein (prM) directly interacts with and activates KDELR1/2-Src signaling to travel on the host secretory pathway [81,90]. Cowpox and measles viruses interrelate with KDELRs via different mechanisms. Cowpox virus evades immunosurveillance by preventing antigen presentation mediated by the class I major histocompatibility complex (MHC-I). In the mildly acidic pH of cis-Golgi, the virus simultaneously binds MHC-I and KDELR, and due to the KDELR retrieval system, brings MHC-I into the ER, where this ternary complex dissociates [84,85]. Conversely, KDELR2 competes with measles virus envelope proteins to bind to ER-chaperones (e.g., calnexin or glucose-regulated protein 78/GRP78), components necessary for the antegrade transport of virus envelope proteins. The overexpression of KDELR2 by increasing the retention of chaperones in the ER causes a reduction in the spread and titer of the virus [91].

Collectively, these observations pose KDELRs as potential therapeutic targets for a broad spectrum of disease conditions.

## 6. KDELRs Are Possible Targets in the Treatment of Diseases, to Improve Drug Delivery and for Immunotherapeutic Production

KDELRs are multifaceted proteins, and although further studies will be required to fully characterize their functions, they can be considered potential targets for the development of novel therapeutic strategies for a variety of diseases. Targeting KDELRs may have beneficial effects in pathologies involving the disruption of proteostasis [40,54,55], in cancer progression [64,65,66,73,74,75,76], as well as in infectious and intoxication diseases by inhibiting the spreading of viral particles and the entry of toxins [78,81,83]. Unfortunately, no pharmacological approach has yet been developed to specifically target KDELRs, with the exception of some drugs, which, by modulating ER-stress and UPR responses, could indirectly involve KDELRs [92,93,94].

However, in recent years, several peptides, antibodies, immunotoxins, and nanoparticles functionalized with the KDEL sequence have been designed and tested in a broad spectrum of diseases. These molecules had different purposes, but all relied on the recognition of their KDEL sequence by the KDELRs. For example, the addition of a KDEL sequence could be used to restrict proteins within the ER. This is the case of the “intracellular sequestration” of the C-X-C chemokine receptor type 4 (CXCR4) enacted by the expression of its ligand, C-X-C chemokine ligand 12 (CXCL12), as a fusion protein with the KDEL peptide. The consequent reduction of the CXCR4 receptor on the plasma membrane decreases metastasis formation of head and neck squamous cell carcinoma [95]. A KDEL-tag trap assay has been proposed to control the secretion of multisubunit proteins with disulfide bridges. Specifically, the secretion of endogenous transforming growth factor beta (TGF-β) family members such as Xenopus nodal-related protein 5 (Xnr5) and bone morphogenetic protein 4 (BMP4) was impaired by expressing one of their subunits fused with a KDEL sequence [96]. In the work by Zhang et al. [97,98], a fusion protein constituted by the human immunodeficiency virus (HIV)-derived transactivator of transcription (TAT) peptide, interleukin 24 (IL-24), and the KDEL motif (TAT-IL24-KDEL) was shown to stimulate apoptosis of large-cell lung carcinoma more effectively than TAT-IL24 or IL24 alone. The peptide TAT-IL24-KDEL permeates the cell membrane due to the TAT peptide and accumulates in the ER thanks to its binding to KDELRs where it induces ER stress and, ultimately, apoptosis [97,98]. The Cholera toxin B (CTB) subunit conjugated to the KDEL sequence (CTB-KDEL) was able to enhance colonic epithelial repair in acute and chronic colitis models. CTB-KDEL, by interacting with KDELRs, collects in the ER and induces UPR, which, in turn, stimulates TGF-beta1/2-production and the migration of colonic epithelial cells, thereby facilitating wound healing. On the other hand, CTB alone or CTB fused to a mutant KDEL sequence had no effect [99,100]. In a recent study, the variable single-chain fragment of anti-human epidermal growth factor receptor 2 (HER2) (4D5) was fused to the ricin A-chain (RTA) protein carrying a KDEL sequence. This novel immunotoxin was efficiently transported within HER2-overexpressing tumor cells and exhibited a strong cytotoxic and anticancer effect compared with RTA-4D5 or RTA alone [101].

Nanoparticles are widely used for drug delivery. The addition of a KDEL motif to gold nanoparticles (AuNP-KDEL) improved their intracellular delivery compared with unconjugated AuNP [102]. In addition, the overexpression of KDELRs further enhances the internalization of AuNP-KDEL constructs. Interestingly, AuNP-KDELs travel through the retrograde transport pathway and accumulate in the ER within minutes, bypassing the degradative lysosomal compartment [102]. AuNP-KDEL has been used to efficiently deliver siRNA against NADPH oxidase 4 (Nox4) in myoblasts and myotubes [103].

A number of vaccine studies have shown that the introduction of a KDEL sequence enhances antigen-specific immune responses by directing KDEL-bearing antigens into the ER, where they have access to the MHC-I presentation pathway, and possibly results in an enhanced and prolonged presentation on the surface of MHC-I/peptide complexes [104,105,106,107,108]. This novel vaccination strategy has been effective in the treatment of virus-dependent tumors such as those induced by the hepatitis C virus [104] or the papillomavirus [105,106,107,108]. For example, human papillomavirus type 16 antigens E6 and E7 fused to the ER signal peptide (SP) and the KDEL sequence resulted in a faster and more efficient antitumor response and increased production of interferon-γ in comparison to SP sequences or E6/E7 antigens alone [107,108].

Finally, an interesting biotechnological application of KDELR-mediated retention within the ER is the preparation of anticancer immunogens and, more generally, the production of recombinant proteins. The inclusion of a KDEL sequence increases both the production and quality of recombinant proteins prepared for therapeutic purposes in plant systems, as reported for the epithelial cell adhesion molecule (EpCAM), one of the tumor-associated antigens [109,110,111]. It has been proposed that the introduction of a KDEL sequence, by retaining KDEL-labeled proteins in the ER, avoids the attachment of complex glycans that could eventually trigger allergic responses in treated patients [111]. In fact, the presence of the KDEL sequence reduced the immunogenicity of these therapeutic molecules.

## 7. Technologies for Assessing KDELR Function

To date, a wide range of functions have been associated with KDELRs, although the list is not exhaustive, and knowledge of the molecular mechanisms involved is still incomplete.

To fill these gaps, we believe that starting from multi-omic approaches, including transcriptomics, proteomics, and metabolomics, in cells with KDELRs activated by trafficking pulses [25,26,29,30], transfected ligands [28,29,30], or exogenous ligands [28,30] may be useful for inferring the role of KDELRs in cell physiology. The same omics approaches should be implemented on cells depleted for or overexpressing individual KDELRs to obtain information on isoform-specific functions. To gain further insight into molecular mechanisms, the indications that will emerge from these system biology approaches will need to be expanded by classical cell biology techniques, including confocal immunofluorescence, fluorescence resonance energy transfer (FRET), immunoelectron microscopy, and coimmunoprecipitations, as described in our recent manuscripts [25,30,112]. Finally, further structural biology investigations are also in demand, with the ideal aim to determine the structure of all three human KDELRs, which would, in turn, be extremely useful both to gain insight into their functional specificity and also to aid in the design of isoform-specific ligands.

## 8. Conclusions

KDELRs function as a sort of gate that rescues ER chaperones and prevents their secretion; this activity is closely linked to ER quality control and the UPR. Furthermore, since the number of chaperones leaving the ER is proportional to the speed of protein synthesis, KDELRs perceive this information and coordinate the anterograde flow of membranes (ER to Golgi and up to the plasma membrane) with the retrograde flow. To accomplish these functions, KDELRs master several signaling cascades.

From a pathological standpoint, these receptors are involved in protein misfolding diseases and cancer, as well as in intoxication and infections. On the other hand, the regulation of KDELR expression and/or activity could be a future therapeutic strategy for a wide range of diseases. Furthermore, they could also be key mediators for novel pharmacological approaches and/or biotechnological elements in the production of biological drugs.

## Figures and Tables

**Table 1:** Pathological conditions associated with KDELRs.

KDELR	Pathological Conditions	Functional Effects	References
KDELRs overexpression	Diabetes	Not yet clarified, probably linked with activation of UPR and MANF interaction	[52,53,59]
KDELRs upregulation/relocated	Neurodegeneration	Increase of protein aggregates Activation of the UPR	[35,40,54,55,56]
KDELR1 mutations	Immune-response	T-cell lymphopenia Defective innate and adaptive immunity	[60,61]
KDELR2 mutations	Osteogenesis imperfecta	Altered collagen secretion that impairs the proper formation of extracellular collagen fibres	[62,63]
KDELRs overexpression	Cancer	Cancer cell proliferation Metastasis Formation	[64,65,66]
